# Medullary Thyroid Carcinoma Mutational Spectrum Update and Signaling-Type Inference by Transcriptional Profiles: Literature Meta-Analysis and Study of Tumor Samples

**DOI:** 10.3390/cancers14081951

**Published:** 2022-04-13

**Authors:** Emanuela Minna, Paola Romeo, Matteo Dugo, Loris De Cecco, Antonella Aiello, Federico Pistore, Andrea Carenzo, Angela Greco, Maria Grazia Borrello

**Affiliations:** 1 Molecular Mechanisms Unit, Department of Research, Fondazione IRCCS Istituto Nazionale dei Tumori, 20133 Milan, Italy; paola.romeo@msn.com (P.R.); loris.dececco@istitutotumori.mi.it (L.D.C.); federico.pistore@istitutotumori.mi.it (F.P.); andrea.carenzo@istitutotumori.mi.it (A.C.); angela.greco@istitutotumori.mi.it (A.G.); 2 Department of Medical Oncology, IRCCS Ospedale San Raffaele, 20132 Milan, Italy; dugo.matteo@hsr.it; 3 Department of Pathology, Fondazione IRCCS Istituto Nazionale dei Tumori, 20133 Milan, Italy; antonella.aiello@istitutotumori.mi.it

**Keywords:** medullary thyroid cancer, transcriptomics, *RET* deletions, genetic landscape, meta-analysis

## Abstract

**Simple Summary:**

Medullary thyroid carcinoma (MTC) is a rare but clinically relevant tumor based on its aggressiveness and the limited therapeutic opportunities currently available for advanced cases. A better understanding of the mechanisms of MTC development is crucial to identify more effective means of intervention and therapies. Several studies have shown that *RET* and *RAS* genes play a central role in MTC. However, little is known about the signaling processes operating downstream of these genes. Here, we report mutation and gene expression profiles in proprietary sporadic MTCs, including both primary and metastatic tumors. We show that tumors derived from the same patient display similar expression profiles and that the latter can be used to obtain information about specific downstream signaling, identifying distinct molecular subtypes. Furthermore, by reviewing the relevant literature, we highlight that, along with *RET* and *RAS*, other less frequent genes are emerging as possible new players in MTC.

**Abstract:**

Medullary thyroid carcinoma (MTC) is a rare but aggressive tumor. Although *RET* and *RAS* genes are recognized drivers in MTC, associated downstream signaling pathways are largely unknown. In this study, we report 17 sporadic MTCs, collected at our institution, comprising patient-matched primary and lymph node metastatic tumors investigated for mutational and transcriptional profiles. As we identified two uncommon *RET* deletions (D898_E901del and E632_L633del), we also performed a literature review and meta-analysis to assess the occurrence of unconventional alterations in MTC, focusing on next-generation sequencing studies. We found that new gene alterations are emerging, along with the known *RET/RAS* drivers, involving not only *RET* by multiple concurrent mutations or deletions but also other previously underestimated cancer-related genes, especially in sporadic MTCs. In our MTC gene profiles, we found transcriptome similarity between patient-matched tissues and expression of immune genes only by a few samples. Furthermore, we defined a gene signature able to stratify samples into two distinct signaling types, termed MEN2B-like and MEN2A-like. We provide an updated overview of the MTC mutational spectrum and describe how transcriptional profiles can be used to define distinct MTC signaling subtypes that appear to be shared by various gene drivers, including the unconventional ones.

## 1. Introduction

Thyroid tissue comprises two distinct types of endocrine cells: follicular cells, which constitute most of the thyroid follicular epithelium and produce thyroglobulin (precursor for the synthesis of thyroid hormones); and parafollicular cells (also called C cells) of neuroendocrine origin, which constitute less than 1% of thyroid tissue and the primary function of which is the secretion of the calcitonin hormone [1].

Thyroid tumors can arise from both cell types, although the vast majority are derived from follicular cells, whereas only 2–3% are derived from C cells and classified as medullary thyroid carcinoma (MTC) [2,3]. C-cell secretory products calcitonin and carcinoembryonic antigen (CEA) are considered MTC markers, and their serum levels are used in MTC patient follow-up [4]. Although quite rare, MTC is an aggressive tumor displaying local invasiveness and metastatic spreading, and few effective treatment options, other than surgery, are currently available for advanced cases [5].

MTC can occur in either hereditary (25%) or sporadic (75%) forms. Hereditary MTCs can develop with different phenotypes, either as familial MTCs or within multiple endocrine neoplasia type 2 (MEN2) syndromes, in which other concurrent malignancies and pathological conditions are observed along with the MTC [6,7]. In these latter, two major subtypes have been described—MEN2 type A (MEN2A) and type B (MEN2B)—displaying both overlapping and distinct clinical features [8]. In particular, a genotype–phenotype correlation has been established, identifying distinctive germline gain-of-function mutations in the tyrosine kinase receptor *RET*, that lead to its aberrant activation. Mutations in the *RET* extracellular domain at various cysteine residues (C609, C618, C620, and C634) have been detected in MEN2A MTC patients, whereas mutations in the *RET* intracellular tyrosine kinase domain (mostly *RET* M918T) have been detected in MEN2B MTC patients [9,10]. Identified in more than 95% of the hereditary forms and in 30–50% of sporadic MTCs [11], *RET* has been recognized as a major driver in this neoplasia.

More recently, *RAS* mutations were detected in a fraction of *RET*-negative sporadic MTCs [12,13], suggesting that other genes can also play a role in MTC tumorigenesis. Also for sporadic cases a different clinical behavior has been observed according with the presence of specific RET mutations, for instance, identifying the highest risk of aggressiveness in RET M918T mutated cases [4].

Although several studies have investigated the mutational background of MTC, only a few have also analyzed the concurrent global gene expression. However, in the latter, the limited number of tested cases, mostly due to MTC rarity, as well as heterogeneous case lists comprising both hereditary and sporadic MTCs harboring various *RET* mutations, have led to confounding results not overlapping across independent cohorts and studies [14], hampering the identification of clear transcriptional profiles related to MTC. Thus, the signaling pathways downstream of recognized oncogenic drivers, as well as whether differences across specific subtypes exist, are still largely unknown in the context of MTC.

In this study we report a series of 17 sporadic MTCs, collected at our institution, including matched primary and lymph node metastatic tumors derived from eight patients.

We have already described this series in a study aimed at the identification of circulating biomarkers for MTC prognosis and treatment response [15]. Here, we report their previously unpublished gene expression profiles.

This series, selected to be representative of the gene drivers identified in MTC [15], includes, along with typical *RET* and *RAS* mutations, uncommon *RET* deletions.

With the aim of assessing whether either these *RET* deletions represent a rare event or whether their occurrence has already been observed in MTC patients, we also performed a literature review. In particular, we focused on the most recent publications applying next-generation sequencing (NGS) in human MTCs. In our review, we also included also studies describing gene expression profiles in human MTC, initially researched as possible independent cohorts for our transcriptomic data validation.

Hereafter, we report the results of our literature review and meta-analysis, providing an updated overview of the MTC mutational spectrum, followed by the transcriptomic data of our MTC samples.

## 2. Materials and Methods

### 2.1. MTC Sample Collection

We investigated 17 tumor tissues derived from 8 sporadic MTC patients, comprising patient-matched primary tumors (*n* = 8) and lymph node metastases (LNMs, *n* = 9) collected during total thyroidectomy and neck dissection. For three patients, LNMs were derived from subsequent lymphadenectomy performed after the initial surgery; for one of them, both a synchronous and a metachronous LNM were available and investigated. Three additional tissues derived from non-neoplastic thyroid (NT) obtained from patients with pathologies other than thyroid cancer were included as controls. Clinical–pathological characteristics for these MTCs are described [15] and summarized in Results Section 3.2.

Genomic DNA from MTCs was screened for *RET* and *RAS* mutations as reported in [15]; both primary tumors and LNMs were assessed, confirming mutational concordance among the patient-matched specimens. *RET* was assessed both at germline and somatic levels to confirm MTC sporadic type.

Furthermore, the following *RET* single-nucleotide polymorphisms (SNPs) were investigated: exon11 rs1799939 (Gly691Ser, G > A), exon13 rs1800861(Leu769Leu, G > T), and exon15 rs1800863 (Ser904Ser, C > G). In detail, DNA was amplified and directly sequenced by the following RET-specific primers (5′–3′ sequence). exon11 forward: CCTCTGCGGTGCCAAGCCTC; exon11 reverse: GACCTGGTTCTCCATGGAGTC. exon13 forward: CAGGAGCGATCGTTTGCAACC; exon13 reverse: ACCTCACCCTGCAGCTGGC. exon15 forward: GACTCGTGCTATTTTTCCTC; exon15 reverse; TATCTTTCCTAGGCTTCCCA.

This MTC set was primarily selected based on: the presence of various *RET*/*RAS* mutations (in order to be representative of MTC gene drivers) and the availability of patient-matched primary/metastatic tumor specimens. As matched specimens were not available for *RET* C634R MTCs, they were not included in the transcriptome analysis.

### 2.2. MTC Study Review and Meta-Analysis

We performed a literature search in PubMed (https://pubmed.ncbi.nlm.nih.gov/; accessed on 15 December 2021), first, to identify studies reporting gene expression profiles in human MTC for our transcriptomic data validation and, second, to assess the occurrence of *RET* deletions in other MTC series. We focused on studies applying high-throughput techniques, such as gene microarrays, RNA sequencing (seq), and NGS. We also included a study using a high-resolution array-based copy number alterations (CNA) platform [16]. The identified studies, along with their major features, are summarized in Table 1.

For each of the literature-derived studies we considered the driving lesions reported for each sample. If the cohort comprised both sporadic and hereditary MTCs, they were assessed as separate groups. If additional MTC cohorts were reported different from that assessed by the specific high-throughput approach (termed “discovery set”), they were also considered (termed “validation set”). A total of 35 MTC cohorts were obtained from literature.

Our MTC series was also assessed; as they derived from a larger cohort [15], for the meta-analysis, we considered the latter, comprising 37 MTCs (33 sporadic and 4 hereditary).

Two studies applying targeted NGS on thyroid cancers, including 8 and 15 MTCs, respectively [17,18] but not providing complete mutation data for each sample, were not included in the meta-analysis.

### 2.3. Gene Expression Profiles in Our MTC Series

Gene microarray profiling was performed on the 17 proprietary, patient-matched MTC samples and 3 NT controls. Total RNA was extracted from FFPE specimens by miRNeasy FFPE kit (Qiagen, Germantown, MD, USA), and its quality was assessed by a Bioanalyzer (Agilent, Santa Clara, CA, USA). Gene profiles were established by HumanHT-12 WG-DASL V4.0 R2 expression beadchip (Illumina, San Diego, CA, USA). RNA labeling, processing, and hybridization were performed according to Illumina standard protocols. Microarrays were scanned with an iScan microarray reader (Illumina, San Diego, CA, USA), and raw data were obtained by iCS software (Illumina, San Diego, CA, USA). Data were processed using the lumi package [19]; raw data were log2-transformed and normalized using robust spline normalization. Multiple probes mapping to the same gene were collapsed using the “maxRowVariance” method [20]. Differentially expressed genes were identified using the limma R package [21]; *p*-values were corrected for multiple testing using the Benjamini–Hochberg false discovery rate (FDR) method, and significance was assigned according to FDR < 0.05 and absolute fold change (FC) > 2 cutoffs. Microarray data were deposited in the NCBI Gene Expression Omnibus (GEO) database (www.ncbi.nlm.nih.gov/geo/; accessed on 7 February 2022) with the accession number GSE196264.

### 2.4. Functional Enrichment Analysis

Gene set enrichment analysis (GSEA) was performed by GSEA version 4.0.1 in pre-ranked mode, using the t-statistic derived from the class comparison analysis of MTC versus NT as ranking metric score. The Hallmarks and the C2 sub-collection Canonical pathway gene sets available in the Molecular Signatures Database v 7.4 were investigated. Gene sets smaller than 15 and larger than 500 genes were filtered out; according to filtering criteria, 50 and 1927 gene sets were tested for the Hallmarks and the Canonical pathways collection, respectively. Gene sets with FDR < 0.05 were considered significant.

For the Hallmarks gene set, functional biological super categories were manually assigned according to the reported “Process Category” [22].

### 2.5. Immune Genes and Scores

The expression of *CTLA4*, *PD-1*(*PDCD1*) and *PD-L1* (*CD274*) genes was assessed in our sample series. We also applied the CIBERSORT [23] and ESTIMATE [24] algorithms that use gene expression signatures to infer the presence of immune cells in tumor specimens. The Immunoscore obtained from ESTIMATE was specifically considered; score data were normalized and represented as Zscore. CIBERSORT *p*-value output was used for sample stratification. *p*-values < 0.05 were considered significant, and samples were assigned to the “positive” for immune infiltration class; whereas *p*-values ≥ 0.05 were assigned to the “negative” class. The list of 18 immune-related genes reported by Pozdeyev et al. [25] termed Immune gene signature (IGS) was also assessed, and a corresponding IGS score was calculated for each sample as mean of the across-sample median-centered expression values.

### 2.6. MEN2B-like and MEN2A-like Signaling Score

First, we investigated the concurrent mRNA expression of a specific gene set previously shown to differentiate MEN2B from MEN2A MTC patients [26]. In detail, an initial list of 118 probes matching 107 genes was derived; genes were reannotated according to the HGNC-approved gene symbol, and nine unmatched genes were filtered out. A final list of 98 genes was obtained, including 82 genes upregulated in MEN2B MTCs and 16 genes upregulated in MEN2A MTCs. The global expression pattern of the 98-gene list was then assessed in our MTC series by unsupervised hierarchical clustering.

Next, an expression score was defined by the quantification of the composite expression of this 98-gene set in each sample. First, two separate scores (an MEN2B and an MEN2A score) were calculated as mean of the across-sample median-centered expression values, considering the 82 MEN2B upregulated genes and the 16 MEN2A upregulated genes separately. Then, a combined MEN2B/MEN2A score was defined, with the purpose of summarizing the information on multiple genes in a single variable. We applied a method analogous to that reported by Rusinek et al. [27]: for each sample, we calculated the sum of expression of the 82 MEN2B upregulated genes minus the sum of expression of the 16 MEN2A upregulated genes; this value was then divided by the number of genes in the signature and centered to the mean across samples. MTCs with a positive score (closer to MEN2B than to MEN2A) were defined as MEN2B-like, whereas MTCs with a negative score (closer to MEN2A than to MEN2B) were defined as MEN2A-like.

### 2.7. MEN2B-/MEN2A-like Score Validation in an Independent Dataset

The public gene dataset GSE32662 (available in GEO) was investigated for MEN2B/MEN2A score validation.

Raw intensity expression values on Agilent Whole Human Genome Array were downloaded (www.ncbi.nlm.nih.gov/geo/query/acc.cgi?acc=GSE32662; accessed on 1 December 2022). Microarray features and raw data were obtained with “getGEO” and “read_maimages” functions of the limma package [21]. Data were background-noise-adjusted and normalized to obtain a log2-expression matrix. Probes not associated to gene symbols and control probes were filtered out. Multiple probes mapping to the same gene were collapsed using the “avereps” function. All analyses were performed using R studio version 4.0.3.

The available gene profiles for the GSE32662 series included 52 MTC samples derived from 49 patients, comprising technical replicates for 3 patients (MTC-3, MTC22, MTC46); for the latter, samples the average expression value between replicates was considered.

The genes related to MEN2B/MEN2A signature, as well as its score definition, were investigated as described above; 94/98 genes were available and were used.

### 2.8. Statistical Analysis

Statistical analyses and significance cutoffs are indicated in the specific subsections. For additional charts and figures, GraphPad Prism v5.02 (GraphPad Software, Inc., San Diego, CA, USA), Clustergrammer [28], or shinyheatmap [29] tools were used.

## 3. Results

### 3.1. Overview of High-Throughput Studies in MTC

First, we reviewed literature reporting gene expression profiles in MTC patients and identified six studies: four applying gene microarrays and two applying RNAseq (Table 1). Among these, only one study [30] displayed transcriptomic data availability (deposition on GEO public repository, series GSE32662) that could be subsequently used as an independent cohort for data validation.

These microarray studies have been recently revised [14]; refer to this publication for a more detailed description of their major findings.

We then expanded our literature review to the most recent publications applying NGS in MTC patients. We identified 12 studies (Table 1, studies 5 to 16; two of them describe both RNAseq and NGS [25,41]) in which either various commercial/custom targeted sequencing panels or whole-exome sequencing (WES) were exploited.

In all these studies, variable tissue specimens (FFPE/frozen), tissue types (primary tumor, metastasis, or not specified (generically indicated as MTC)), and MTC subtypes (sporadic, hereditary, or unknown) were investigated.

Excluding the set from Ciampi et al. [11], which represents the largest cohort investigated in MTC by a high-throughput approach to date, only three studies were able to collect samples from more than 45 MTC patients.

Collectively, considering both transcriptional and NGS approaches, 16 independent studies were found in the literature. Their major features are summarized in Table 1, along with those of our MTC series (study number 17).

### 3.2. Meta-Analysis of MTC Gene Drivers Highlights a More Complex Mutational Landscape

For each of the abovementioned studies (Table 1), we examined the driving lesions reported in each sample and their distribution across MTC subtypes.

Considering MTC subtypes, along with the sporadic and the hereditary types, an additional group termed “unknown” was found, including samples for which the sporadic/hereditary definition cannot be assigned due to unavailable data for *RET* germline mutational status and/or for MTC patient family history. The “unknown” samples identified in three different studies were analyzed as a separate group.

Along with the above-described case lists (Table 1), some studies reported additional MTC cohorts assessed, for instance, as validation set [25,30,31,38,41], which were also examined. Regarding our sample series, for the meta-analysis, we considered the entire cohort of 37 MTCs, from which the 17-MTC subset had been selected [15].

**Figure 1:**
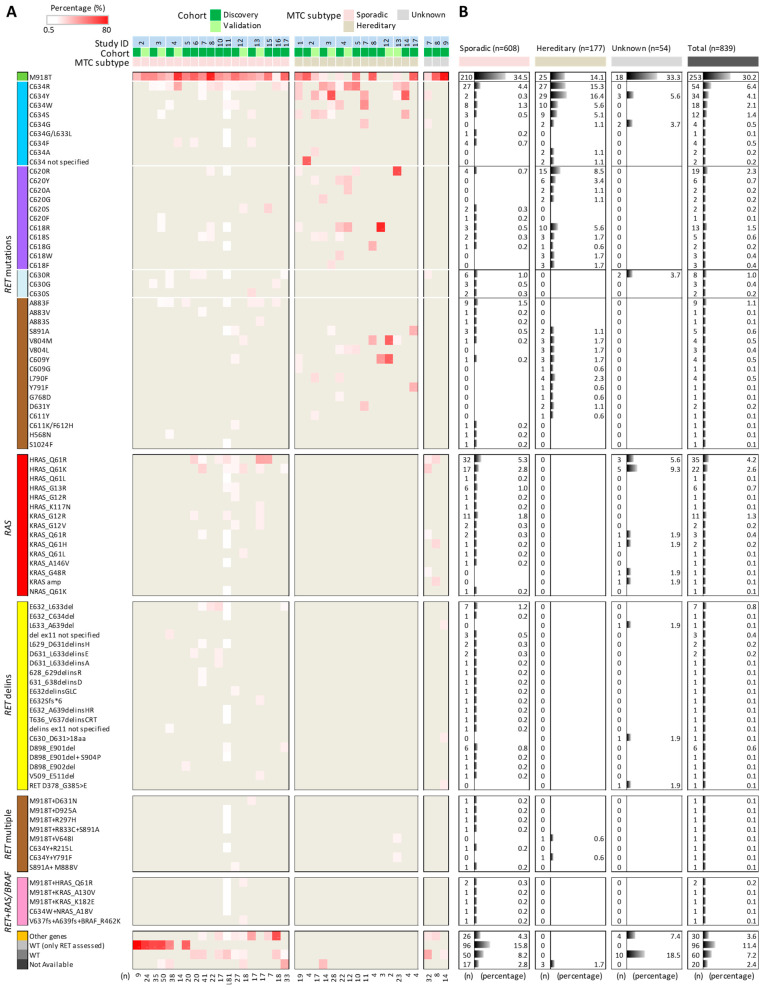
Meta-analysis of MTC patient mutational spectrum. (**A**) Mutation types identified in the 37 MTC cohorts derived from the 17 studies reported in Table 1. Cohorts are ordered based on MTC subtype and study. Heatmap shows the percentage of positive samples for the specific mutation in each cohort; the total number of samples (*n*) composing each cohort is at the bottom. For our series (study 17), the original cohort of 37 MTCs was considered. (**B**) Frequencies of the corresponding mutations in the specific MTC subtypes and in the whole set (total); the total number of mutation-positive patients (*n*) and their percentage are shown.

Collectively, 37 cohorts derived from 17 independent studies were identified and investigated, for a total of 839 MTC cases stratified in 608 sporadic, 177 hereditary, and 54 unknown samples (Figure 1).

Most of the reported genetic alterations, as expected, involve *RET* and *RAS* genes (Figure 1A). The most frequently mutated hotspot is confirmed to be *RET* M918T, followed by *RET* C634, with different amino acid substitutions; R and Y are the most prevalent and are especially detected in the hereditary cases, where they represent the prevalent driving lesions (C634R 15.3% and C634Y 16.4%, followed by M918T 14.1%; Figure 1B). In hereditary cases, mutations involving the C620 and C618 codons (with R substitutions being the most common) are also observed in multiple samples and cohorts, although they are quite infrequent in sporadic cases. Similarly, mutations involving codons C609, C611, D631, G768, L790, Y791, and V804 are observed almost exclusively in hereditary cases, although with variable percentages across the different cohorts. In contrast, mutations involving C630 and A883 codons, as well as the less common H568 and S1024 codons, appear to be specific to sporadic cases.

Following these *RET* mutations, frequent point mutations are also observed in *RAS* genes that represents the second top driver in MTC, especially *H/KRAS* (codons Q61 and G12/13 as the most common hotspots). *RAS* mutations appear to be specifically detected in sporadic cases and are completely absent in hereditary cases. Similar sporadic-specific distribution was also observed for MTC unconventional genetic alterations as (i) small *RET* deletion (del) or deletion coupled with insertion (delins), (ii) multiple co-occurring *RET* mutations, and (iii) mutations in multiple genes.

Our meta-analysis shows how *RET* del/delins, although rare within a given case list (detected in very few samples), are consistently reported by a progressively increasing number of independent studies (collectively, 5.3% of 609 sporadic cases; Figure 1B). The most frequent *RET* del/delins involve exon11 in the neighborhood of codons from 628 to 639 with variable alteration pattern, with the E632-L633del as the most common, followed by the D898-E901/E902del affecting *RET* exon15. Alternative *RET* del/delins, such as 509–511del and other insertions, have also been reported [36].

Multiple co-occurring *RET* mutations and mutations involving both *RET* and other MAPK pathway effectors, such as *RAS* and *BRAF*, have also been detected, although most have only been detected in a single study [11]. The only exception is represented by the *RET* M918T + *HRAS* Q61R double mutation described in two independent studies [11,25]; interestingly, both studies used a targeted NGS panel for thyroid-specific genes.

Furthermore, our meta-analysis confirms that studies applying bigger NGS panels or WES are able to identify possible drivers other than *RET* and *RAS* genes and to reduce the proportion of WT cases (those with unknown gene drivers) compared to earlier studies that assessed only *RET* gene mutations (Figure 1A, study from 2 to 5). Interestingly, WT samples, as well as those with mutations in “other genes”, are observed essentially only among the sporadic cases, whereas most of the hereditary cases appear to be driven by a specific set of *RET* mutations (*RET*-positive cases account for 98.3% of the 177 hereditary cases; Figure 1B).

For the “unknown” samples (those for which the sporadic/hereditary type cannot be assigned; Figure 1A), our analysis reveals the occurrence of gene alterations found in both sporadic and hereditary cases, such as *RET* M918T (33.3%) and *RET* C634 (9.3%), as well as alterations specifically observed in sporadic cases, such as *RET* C630R (3.7%), *RET* delins (5.5%), and *RAS* mutations (22.2%). In addition, this “unknown” group also includes specifically identified MTC unconventional alterations (such as KRAS_G48R mutation, KRAS amp, and particular RET insertions), the significance of which remains to be explored.

**Figure 2:**
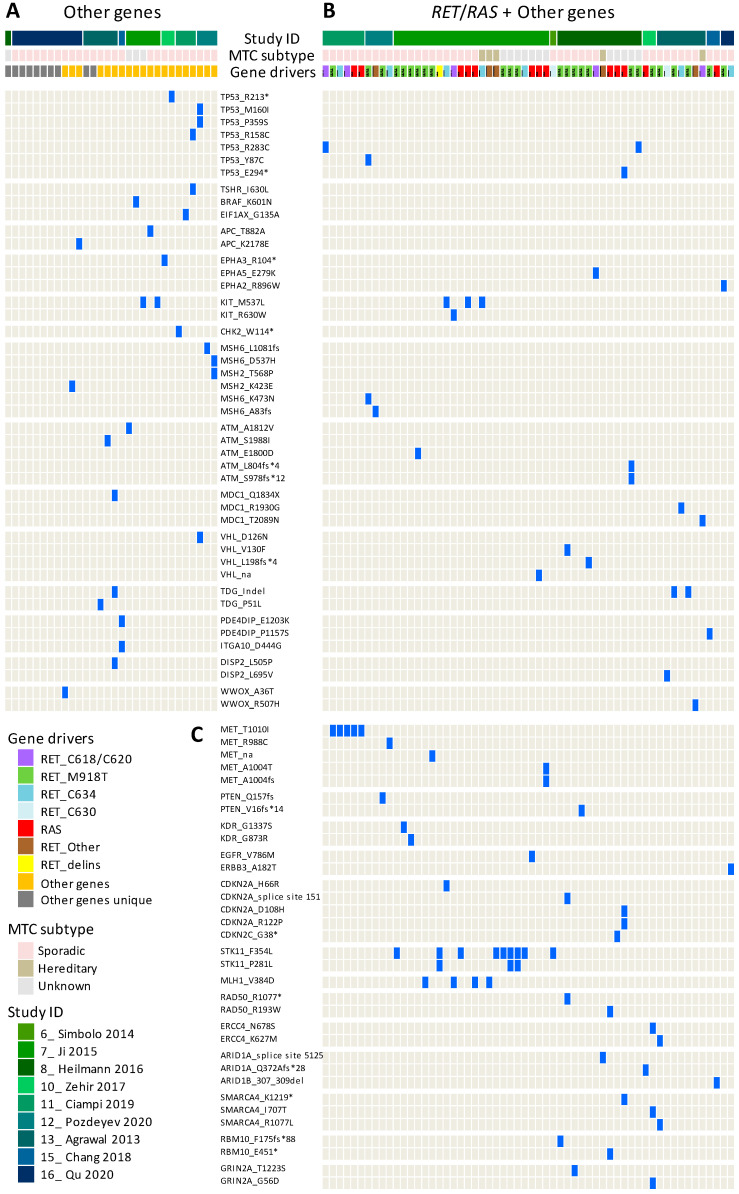
Mutations in “other genes” in MTC patients. The type and distribution of mutations affecting other genes different from *RET* and *RAS* is shown. Each column represents a specific patient, and the presence of the indicated mutations is indicated by a blue tag. Only genes mutated in at least two patients are shown. (**A**) Patients lacking *RET/RAS* mutations (*n* = 30) were assigned to the group “other genes”; patients with mutations in other genes not identified in at least two patients were assigned to the group “other genes unique”. (**B**) Patients with concurrent *RET/RAS* mutations (*n* = 58). (**C**) Genes mutated only in patients reported in (**B**). * indicates nonsense mutations.

Among cases negative for *RET/RAS* alterations (group termed “other genes”; Figure 1), mutations in other cancer-relevant genes have been reported (Figure 2A), such as in the tumor suppressors *TP53* and *APC*, known thyroid-cancer-related genes (*TSHR*, *BRAF*, and *EIF1AX*), tyrosine kinase receptors (*EPHA3* and *KIT*), DNA damage response genes (*CHK2*, *MSH6*, *MSH2*, *ATM*, and *MDC1*), and other less common genes. Mutations in these genes are found in multiple patients, although the type of mutation appears quite heterogeneous and mostly patient-specific. Mutations in these genes are also identified in patients harboring *RET*/*RAS* mutations (Figure 2B), suggesting that they may not be mutually exclusive with respect to these known gene drivers. Even considering these additional genes, a proportion of samples, especially those from the Qu et al. study [41], still remains, with an unknown driver (termed “other genes unique”), as they harbor mutations in unique genes not shared by other patients, the role of which remains to be fully characterized (Figure 2A).

Mutations in additional genes belonging to similar pathways are then specifically observed only in *RET*/*RAS*-mutated patients and affect, for instance, the tumor suppressor *PTEN*, other tyrosine kinase receptors (*MET*, *KDR/VEGFR2*, *EGFR*, and *ERBB3/HER3*), and DNA damage response genes (*MLH1*, *RAD50*, and *ERCC4*), as well as cell cycle regulators (*CDKN2A* and *CDKN2C*) and SWI/SNF chromatin remodeling components (*ARID1A*, *ARID1B*, and *SMARCA4*) (Figure 2C).

Collectively, alterations in multiple genes are observed in 11% of the cases (94/839 MTCs) if we consider samples with mutations in “other genes” (*n* = 30, Figure 2A) alone or associated with *RET/RAS* drivers (*n* = 58, Figure 2B) and samples with *RET+RAS/BRAF* mutations (*n* = 6, Figure 1). The co-occurrence of multiple alterations in these “other” genes (Figure 2) has been similarly observed in aggressive thyroid tumors of follicular-cell origin [42,43,44]. Thus, the finding of multiple alterations in MTC, although in a fraction of cases, indicates that in this tumor type, the mutational landscape could also be more complex than previously thought.

Focusing on our cohort (37 MTCs, including 33 sporadic and 4 hereditary cases; Figure 1, study 17), we found a mutational panel consistent with the other cohorts, including, along with common *RET* mutations, two of the most frequent *RET* deletions (i.e., E632-L633del and D898-E901del); for a discrete fraction of samples (27%), unfortunately, mutational data were not available due to low-quality FFPE-derived DNA.

**Figure 3:**
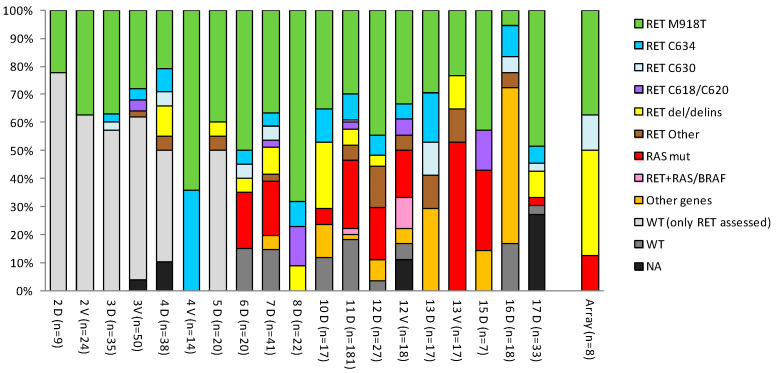
Mutational spectrum in sporadic MTC cohorts. The percentage of the mutations reported in each MTC sporadic cohort is shown; cohorts are identified by the corresponding study ID (see Table 1) and the discovery (D) or validation (V) set type. The number of samples (*n*) in each cohort is in parentheses. The “Array” bar represents the eight patients described in Table 2 selected from a larger cohort (study 17). The *RET* Other class includes both mutations in less frequent codons and multiple co-occurring *RET* mutations.

The subset of eight sporadic MTC patients (Table 2) selected from this larger cohort, the gene expression profiles of which are hereafter described, although small, displayed a mutational pattern similar to that of the other sporadic MTC cohorts (Figure 3), particularly the most recent cohort including both *RAS* mutation and *RET* deletions.

### 3.3. Gene Expression Profiles in Our Cohort of Sporadic MTCs Identify Specific Sample Stratification and Signaling Pathways

Gene expression was assessed by microarray in the above-described set of sporadic MTCs (Table 2), comprising patient-matched primary tumor/LNM and NT controls.

**Figure 4:**
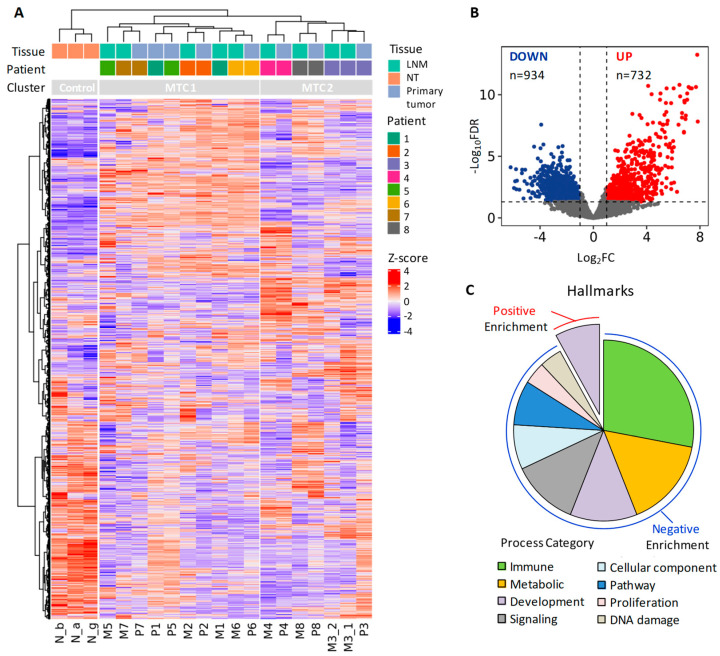
Gene expression profiles in our sporadic MTC cohort. (**A**) Unsupervised hierarchical clustering according to the top 2000 most variable genes in the proprietary series of 17 sporadic MTCs (patient-matched primary tumors and lymph node metastases (LNMs)) and 3 non-neoplastic thyroid (NT) controls. (**B**) Volcano plot of differentially expressed genes in MTC compared to NT. Log2 fold change (FC) and −log10 false discovery rate (FDR) values are shown; dashed lines represent cutoffs for differentially expressed gene selection (absolute FC > 2 and FDR < 0.05). Up- and downregulated genes are shown in red and in blue, respectively. (**C**) Distribution of the significant (FDR < 0.05) Hallmarks process categories from GSEA relative to the gene set shown in (**B**); see Figure S1C for Hallmarks collection complete data.

As expected, NTs displayed transcriptional profiles different from those of MTCs, forming a separate group in unsupervised analyses (Figure 4A, hierarchical clustering, and Figure S1A, principal component analysis).

Regarding MTCs, we did not observe sample stratification based on tissue type (either primary tumor or LNM) (Figure 4A and Figure S1A); however, we found a patient-specific stratification, with matched specimens clustered together (Figure 4A), with only the exception of two metastatic samples. This observation is consistent with previous reports describing significant transcriptome similarity between thyroid-cancer-derived primary tumors and patient-matched metastases, especially the LNMs [45,46,47].

This was further confirmed by class comparison analysis. Whereas a consistent panel of gene deregulation was identified in MTCs vs. NTs (Figure 4B, considering all 17 MTC samples) and further confirmed in primary tumors vs. NTs (Figure S1B), in the LNMs vs. primary tumors comparison, only two genes were differentially expressed with statistical significance (Figure S1B). The first was the thyroglobulin (TG) gene, a known marker of a normal thyroid follicular epithelium, and the second gene (MAB21L2) was also found to be overexpressed in normal thyroid [48].

Both of them were downregulated in LNMs (Figure S1B), consistent with their different derivation site (LNMs derived from neck lymph nodes and primary tumors derived from thyroid). Thus, their differential expression can be explained by the presence of few residual normal thyroid cells in the primary tumor specimens.

Focusing on MTC vs. NT genes (Figure 4B and Table S1), among the top downregulated genes we found that genes related to thyroid function (such as *TG*, *FOXE1*, *SLC26A4*, *TSHR*, *TPO*, *GLIS3*, *DUOX1*, *DUOX2*, *PAX8*, and *DIO2*) were highly expressed in the NTs, as expected [49]. In contrast, among the top upregulated genes, we found MTC/C cell-specific markers, such as CEA (*CEACAM5* gene and its paralog, *CEACAM6*), calcitonin (*CALCA* gene and its paralog, *CALCB*), and genes related to neuroendocrine functions (*CHGA*, *CHGB*, and *GRP*), in agreement with previous reports [25,26,31], as well as the *RET* gene and its coreceptor, *GFRA4*, in agreement with its oncogenic activation.

To better understand the biological processes represented by this gene panel (Figure 4B), we next performed gene set enrichment analysis (GSEA). First, we explored the Hallmarks gene set collection and identified several pathways significantly enriched in MTC compared to NT (FDR < 0.05; Figure 4C and complete list in Figure S1C). Most of them were associated with gene downregulation (negatively enriched pathways), mainly related to immune, metabolic, development, and signaling functions. Only two pathways were positively enriched in MTC vs. NT, involving functions related to development (Figure 4C) and, in particular, endocrine-system-related organs (Figure S1C).

As the Hallmarks represents a general and concise collection [22], we then performed GSEA using the C2-Canonical pathways collection to explore more specific and detailed pathways (Figure S1D). This second analysis not only confirmed Hallmarks findings about the negatively enriched pathways in MTC involving immune-related and metabolic processes but also revealed the positive enrichment of neuroendocrine processes and pathways involving specific neurotransmitters (Figure S1D and complete list in Table S2).

As GSEA results suggested the downregulation (negative enrichment) of immune pathways in our MTC samples, to further confirm these data, we then investigated specific immune-related genes and signatures.

**Figure 5:**
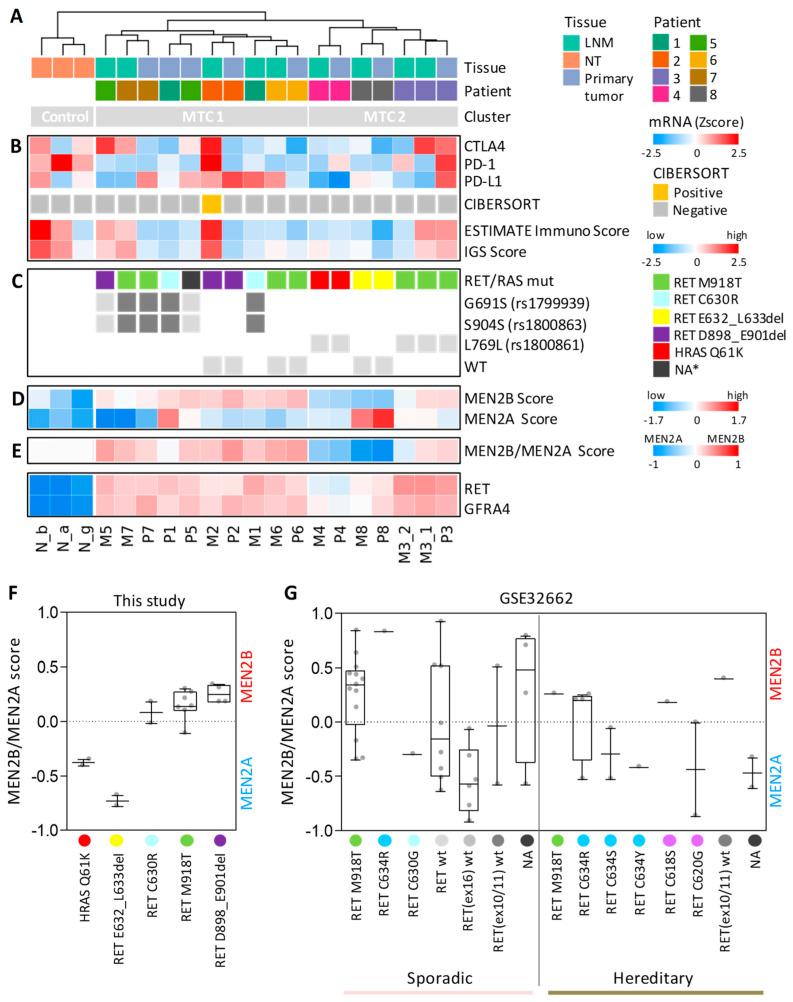
Assessment of immune- and MTC-related gene signatures in our sporadic MTC cohort. (**A**) Unsupervised hierarchical clustering as reported in Figure 4A. LNM, lymph node metastasis; NT, non-neoplastic thyroid. (**B**) Expression of immune-related genes and signatures. CIBERSORT class attribution according to *p*-value (<0.05, positive; ≥0.05, negative). ESTIMATE Immunoscore expressed as normalized data (Zscore). Immune gene signature (IGS) score calculated as mean expression of the 18 IGS genes. (**C**) *RET/RAS* mutational status. NA *, not available data (this sample is the matched specimen for M5 LNM-harboring *RET* D898_E901del). Specific *RET* SNPs or their absence (WT) is indicated in grey. Dark grey indicates homozygosis. (**D**) Gene expression scores calculated separately for MEN2B and MEN2A upregulated genes, respectively. (**E**) Combined score for the attribution of either MEN2B-like or MEN2A-like type based on MEN2B/MEN2A signature. (**F**) MEN2B/MEN2A score in our sporadic MTCs stratified for driving lesion. All the specimens (primary tumor and LNM) are shown for each patient. (**G**) MEN2B/MEN2A score in the public MTC GSE32662 series stratified for MTC subtype and driving lesion.

We assessed the expression of *CTLA4*, *PD-1*, and *PD-L1* genes in our sample series (Figure 5B) and found average low expression of *CTLA4* and *PD-1* in MTCs compared to NTs, whereas *PD-L1* expression was more heterogeneous across samples (Figure S2A). Only 2 out of 17 MTC samples (11.8%) displayed concurrent high levels of the three genes (M2 and P3; Figure 5B).

We also used CIBERSORT [23] and ESTIMATE [24] algorithms, able to predict the immune cell infiltration in tissue specimens by testing specific immune-related gene signatures. Among our samples, CIBERSORT identified only the LNM sample M2 as positive for the presence of immune infiltration (*p*-value < 0.05, Figure 5B). ESTIMATE produced similar although less stringent results, identifying a high immune score in few additional samples. As Pozdeyev et al. reported the enrichment of an immune gene signature (IGS) in a subset of MTCs, we also tested this gene set [25]. A total of 9 out of 18 genes composing the IGS were included in the immune gene set used by ESTIMATE. We observed overlapping results between IGS signature and ESTIMATE Immunoscore (Figure 5B).

Collectively, we found that only a few samples in our MTC series displayed immune infiltration. Although limited by the small size of our MTC cohort, these data are in agreement with previous reports describing immune infiltration in only small subsets of MTC patients [50,51].

### 3.4. MTC Signaling Subtypes Are Inferred by an MEN2B/MEN2A-Related Gene Signature

Considering the unsupervised hierarchical clustering in our samples, we observed an MTC stratification in two major clusters (MTC1and MTC2) not related with either the tissue type or the possible presence of immune infiltration (Figure 4A and Figure 5A,B).

As our MTCs harbor various *RET/RAS* mutations (Table 2), we investigated whether their stratification could be affected by different driving lesions. In particular, we wanted to test, first, whether *RET* M918T patients (3/8 of our series) formed a homogeneous group compared to the other patients and, second, whether patients with different *RET* deletions (D898_E901del and E632_L633del) displayed similar transcriptomes.

We found that *RET* M918T patients only partially clustered together (patients 6 and 7 in MTC1 and patient 3 in MTC2; Figure 5C). All patient 3 samples, clustering separately, displayed a low *RET* M918T mutational frequency [52].

Then, we found that *RET* D898_E901del samples clustered together in the MTC1 group, whereas *RET* E632_L633del clustered in the MTC2 group, along with *RAS*-mutated samples.

In addition, we observed a different distribution of *RET* polymorphisms. Samples with rs1799939 (G691S) and rs1800863 (S904S) SNPs in our MTCs detected in cooccurrence either in heterozygosis or in homozygosis localized in the MTC1 group, whereas samples with rs1800861(L769L) SNPs localized in the MTC2 group (Figure 5B).

Thus, the observed MTC stratification (MTC1 and MTC2 clusters) can be explained only in part by the specific type of mutations.

Next, we tested an MTC-related gene set previously described to differentiate MTC patients with MEN2B (all harboring *RET* M918T) from those with MEN2A (harboring various *RET* mutations: C609, C618, C620, and C634) [26].

We first assessed the global expression of this MEN2B/MEN2A signature (Figure S2B) and observed consistent sample stratification, confirming NT separation, clustering of patient-matched specimens, and separation of *RET* E632_L633del- and *RAS*-mutated samples, as well as separation of *RET* M918T-diverging samples from patient 3.

With the aim of combining the total gene information into a single variable, we then defined an expression score based on this gene signature. Initially, an MEN2B score and an MEN2A score were established, separately considering the genes upregulated in MEN2B and in MEN2A, respectively. We found that both NT- and *RAS*-mutated MTCs displayed low scores (Figure 5D), indicative of average low expression of both gene sets. A high MEN2A score was specifically observed in *RET* E632_L633del samples, whereas the other samples displayed a high MEN2B score. One sample with *RET* C630R (primary tumor from patient 1; Figure 5D) displayed high concurrent expression of both scores.

We then focused on MTCs and defined a combined MEN2B/MEN2A score, summarizing separate score information and the corresponding MEN2B-like or MEN2A-like type. We found that samples in the MTC1 cluster were MEN2B-like type, with the exception of the P1 sample, which was neutral, in agreement with the observed expression of both MEN2B and MEN2A gene sets (Figure 5D). In contrast, samples in the MTC2 cluster were MEN2A-like. The *RET* M918T-diverging samples from patient 3, although with a lower score, were MEN2B-like type, in agreement with the other *RET* M918T samples.

Collectively, we found different signaling subtypes in our MTC set based on MEN2B/MEN2A gene signature. The latter are consistent across patient-matched specimens and various mutations, as well as with the observed MTC unsupervised clustering. In particular, *RET* M918T samples were MEN2B-like (Figure 5F), with only one exception (M3_2 metastatic sample), as well as *RET* D898_E901del samples, whereas *RAS*-mutated and *RET* E632_L633del samples were MEN2A-like. *RET* C630R samples displayed a particular borderline type.

As validation for MEN2B-/MEN2A-like signaling types, we explored the only other MTC dataset found in the literature with publicly available transcriptomic profiles (GEO series GSE32662; see Table 1). This set comprised 49 patients, including both sporadic and hereditary MTCs partially characterized for *RET* mutations (Figure 5G). Although sparse across various MTC and mutation types, with specific categories represented by only one or few samples, we found coherent data about MEN2B-/MEN2A-like types.

For the most part, *RET* M918T samples were MEN2B-like (11/14, 78,6%), including both sporadic and hereditary (one) samples, whereas other *RET* mutations were found to be either MEN2B- or MEN2A-like type, similarly to samples with an unknown driver (*RET* wt, *RET* only partially assessed, or not available -NA- data). Interestingly, all sporadic samples negative for the presence of *RET* M918T (i.e., wt for *RET* exon16) were MEN2A-like.

Of note, different MEN2B-/MEN2A-like types were observed in relation to the same *RET* mutation but with different amino acid substitution. In particular, referring to C634 residue, Arg (R) was associated with MEN2B-like type (4/5 of mutated samples), whereas either Ser (S) or Tyr (Y) substitutions were associated with the MEN2A-like type. Similarly, referring to C630 residue, R substitution was associated with the MEN2B-like type in our samples (Figure 5F), whereas Gly (G) substitution was associated with the MEN2A-like type in the GSE32662 sample (Figure 5G). This possibly suggests that substitutions by amino acids harboring specific chemical and structural features may differently impact downstream signaling and related subtypes. However, these represent preliminary observations that require further studies and validation in additional samples and cohorts.

Collectively, these data suggest that the application of MEN2B/MEN2A signature and its derived score can be used to infer signaling subtypes in MTC.

## 4. Discussion

In this study, we reported gene expression profiles in a sporadic MTC series, comprising, along with typical *RET/RAS* mutations, patients with uncommon *RET* deletions.

Through a literature review and meta-analysis, we provided evidence that *RET* deletions, as well as other uncommon genetic alterations, are identified in MTC by a progressively increasing number of studies, in particular, in those applying next-generation sequencing.

Our study highlights how, along with the known and well characterized MTC gene drivers, additional less frequent genetic alterations are emerging. Although rare and detected in few samples, they are consistently identified in several independent cohorts. They involve not only the *RET* gene, in which single and multiple point mutations, as well as small deletions/deletions–insertions are described, but also other cancer-related genes, the alteration of which has been reported either alone or in association with the *RET*/*RAS* drivers. Although these other cancer-related genes (such as TP53, tyrosine kinase receptors, DNA damage response effectors, etc.) are well characterized in other tumor types, including follicular-cell-derived thyroid cancers [42,43,44], in the MTC context, their identification represents a recent finding, and their role remains to be further investigated and defined.

A step toward a better understanding of the downstream effects associated with these unconventional genetic alterations in MTC can be represented by the results described herein concerning *RET* deletions.

Three patients harboring the two most frequent *RET* deletions found in MTC (i.e., exon11 E632-L633del and exon15 D898-E901del) were investigated herein. For these samples, we observed different transcriptional profiles and signaling types, with E632-L633del samples resembling MEN2A (prevalently characterized by *RET* exon11 mutations), whereas D898-E901del samples resemble MEN2B (signaling type identified in *RET* exon16 M918T-mutated patients).

This indicates that different *RET* deletions activate distinct signaling cascades consistent with their localization in *RET*-specific functional domains (i.e., the extracellular cysteine-rich domain for exon11 lesions and the intracellular tyrosine kinase domain for exon15–16 lesions, respectively).

To the best of our knowledge, only two other studies have reported transcriptional profiles of MTCs with *RET* deletions. One study described a sporadic MTC with *RET* E632delinsGLC (study 12, Table 1), for which only the expression of specific immune- related genes was reported [25].

Another study described four sporadic MTCs with unspecified *RET* exon11del/delins (study 4, Table 1); however, their expression profiles are not clearly identifiable, as they were anonymously reported, along with other sporadic/hereditary MTCs harboring various *RET* mutations [32].

Interestingly, the authors of the latter study also proposed an MTC sub-classification termed MEN2B-like and MEN2A-like, although this was assigned a priori based on the *RET* mutation type (MTCs with *RET* exon10-11 mutations assigned to the MEN2A-like class, whereas MTCs with *RET* exon16 M918T mutation assigned to the MEN2B-like class) [32]. In contrast, in the present study, we derived our MEN2B-/MEN2A-like signaling type in an unbiased manner based on the composite expression of a 98-gene panel previously identified in MEN2B vs. MEN2A MTCs [26]. This classification can be applied, as our data showed, not only to patients with canonical *RET* mutations (exon10-11 and exon16) but also to patients with uncommon genetic alterations or even with unavailable mutational data to infer their signaling type and obtain a clue about their resemblance to an MEN2B or a MEN2A MTC.

Oczko-Wojciechowska et al. also reported a four-gene classifier comprising two genes (NNAT and PTPRT) upregulated in MTCs with MEN2A-like mutations and two genes (GABRR1 and NTRK3) upregulated in MTCs with MEN2B-like mutations [32]. Two of these genes are also included in our MEN2B/MEN2A signature (Table S3; NNAT in the MEN2A upregulated gene set; GABRR1 in the MEN2B upregulated gene set). This four-gene classifier displays mostly coherent expression in our MTCs and consistency with our sample classification (Figure S3A), suggesting that overlapping and combinable results can be obtained. Unfortunately, we cannot assess our MEN2B/MEN2A signature in their MTC series, as transcriptomic data are not deposited and not available for independent testing.

Furthermore, we found that the expression of another gene, *PROM1*, indicated as overexpressed in *RET* M918T-mutated MTCs [30], is consistent in our samples and with our MEN2B/MEN2A scoring (Figure S3B).

This collectively suggests that our MEN2B-/MEN2A-like classification not only significantly correlates with relevant genes identified by other MTC studies but that it could also be integrated and possibly optimized in the future with the addition of further gene markers identified in MTC.

A critical point in the MTC research field is represented by the lack of specific gene markers differentiating MTC from its non-tumoral control.

In this study, as in others [34,53], we considered a normal thyroid as non-neoplastic control. Nevertheless, we are aware that this represents a limitation because the proper non-tumoral control for MTC should be endocrine C cells, although this is currently not feasible. C cells constitute less than 1% of the normal thyroid and are interspersed throughout the tissue; their isolation, collection, and purification are still difficult to achieve. To our knowledge, only a few attempts have been made in this regard, essentially based on cell sorting in non-human tissues [54], with limited results and no further investigations. Thus, this still remains an issue to be resolved.

It is possible that future technologies, such as single-cell RNA sequencing or spatial transcriptomics, could be exploited to address this point. The application of these emerging advanced technologies able to resolve gene expression information at the single-cell level [55] could produce useful data for the rare subpopulation of C cells, leading to the identification of which genes and pathways are physiological in these endocrine cells and which are relevant to MTC oncogenic transformation.

In addition, another investigation useful to better understand the MTC transformation and progression cascade could be the analysis of larger MTC case lists comprising both early and late-stage MTCs. This could provide information about the possible processes involved in MTC cell transformation toward a more aggressive phenotype.

In our unsupervised clustering, we observed that, along with the driving lesion and the MEN2B-/MEN2A-like transcriptional subtype, the presence of specific *RET* polymorphisms could also possibly contribute to MTC sub-classification. This is in agreement with previous reports describing that specific *RET* SNPs may act as genetic modifiers in MTC, possibly affecting its susceptibility, progression, or phenotypic expression [56,57,58], although controversial data exist regarding this issue [59]. A possible affecting role of *RET* SNPs therefore needs to be confirmed in additional samples and cohorts; however, due to MTC rarity, such cohorts are difficult to obtain.

Our literature review further highlights how, excluding Ciampi et al., set [11], most of the tested cohorts, including ours, comprise a small number of patients, confirming the difficulties for individual research groups to collect large MTC sets.

In this sense, a common initiative for the collection of MTC case lists derived from multiple centers and their genomic characterization by integrated high-throughput approaches should be seriously taken in consideration. However, to avoid variable quality of the input material and of the derived results, standard operating procedures should be applied for sample collection and storage. To this end, the creation of a consortium networking multiple centers could provide guidelines for pre-analytical sample processing and identification of a few accredited laboratories for omics data generation according to a centralized approach. Similar initiatives have already proven successful, as observed for consortia such the Cancer Genome Atlas.

Another point to be taken in consideration is the general concept of data usability and sharing, such as transcriptomic data deposition, as this represents an essential tool for data validation, especially in the context of rare diseases, such as MTC.

## 5. Conclusions

In this study, we provided an updated overview of the mutational spectrum identified in MTC. We highlighted how, especially in sporadic MTC, new gene alterations involving both *RET* and other cancer-related genes are emerging alongside the well-known *RET* and *RAS* mutations, suggesting a more complex mutational landscape than previously thought.

We also described how, along with driving lesions, additional transcriptional and genetic elements, such as expression of distinct gene sets and the presence of specific *RET* polymorphisms, could differently affect MTC oncogenic pathways.

The application of an MTC-related gene set led to the definition of two alternative signaling types, indicated as either MEN2B-like or MEN2A-like. These signaling types are identified both in the presence of canonical drivers, such as *RET* M918T and *RAS* mutations, and in the presence of rare alterations, such as *RET* deletions. Particularly for MTCs with uncommon alterations, this represents a useful finding to better understand the downstream signaling mediated by these less frequent and still poorly investigated lesions.

## Figures and Tables

**Table 1:** List of studies investigating MTC patient cohorts with high-throughput approaches.

N	Reference	Approach	Platform	Subtype *	Specimen *	Tissue *	Patient (*n*) *	Sample (*n*) *	Data Deposition **
1	Jain 2004 [26]	Microarray	Hg-U95Av2 GeneChips (Affymetrix)	H	Frozen	P/M	19	25 ^1^	No
2	Ameur 2009 [31]	Microarray	Custom-designed (Agilent Technologies)	S/H	Frozen	P	13	13	Yes (arrayexpress) ^2^
3	Maliszewska 2013 [30]	Microarray	Whole Human Genome Array 4 × 44K (Agilent Technologies)	S/H	Frozen	MTC ^3^	49	52 ^4^	Yes (GSE32662)
4	Oczko-W. 2017 [32]	Microarray	GeneChip Gene 1.0 ST arrays (Affymetrix)	S/H	Frozen	MTC ^3^	60	60	No
5	Ye 2008 [16]	CNA	Human Genome hybridization 244K platform (Agilent Technologies)	S/H	Frozen	P	30	30	No
6	Simbolo 2014 [33]	Targeted NGS	Ion AmpliSeq Cancer Hotspot Panel v2 (Life Technologies) ^5^	S	FFPE	MTC ^3^	20	20	No
7	Ji 2015 [34]	Targeted NGS	Ion AmpliSeq Cancer Hotspot Panel v2 (Life Technologies) ^5^	S/H/U ^6^	FFPE	MTC ^3^	84	84	No
8	Heilmann 2016 [35]	Targeted NGS	FoundationOne panel ^7^	S/H/U ^8^	FFPE	P/M ^8^	34	34	No
9	Vanden Borre 2017 [36]	Targeted NGS	FoundationOne panel ^7^	U ^9^	FFPE	P/M ^9^	14	14	No
10	Zehir 2017 [37]	Targeted NGS	MSK-IMPACT panel ^10^	S ^11^	FFPE	P/M ^11^	17	17	Yes (cBioportal) ^10^
11	Ciampi 2019 [11]	Targeted NGS	Custom panel (Thermo Fisher) ^12^	S	Frozen/FFPE	P/M ^13^	181	181	Yes (COSP47106) ^12^
12	Pozdeyev 2020 [25]	Targeted NGS; RNAseq	ThyroSeq v3 ^14^, TruSEQ RNA Exome (Illumina)	S/H	FFPE	P/M ^15^	27	30^15^	No
13	Agrawal 2013 [38]	WES	SureSelect paired-end v2.0 human exome (Agilent Technologies)	S	Frozen	MTC ^3^	17	17	No
14	Cai 2015 [39]	WES	44M human exome array (NimbleGenEZ)	H	Frozen	MTC ^3^	4	4	No
15	Chang 2018 [40]	WES	TruSeq Exome (Illumina)	S	NA	MTC ^3^	7	7	No
16	Qu 2020 [41]	WES; RNAseq	AI whole Exome CNV (iGeneTech); VAHTS RNA-Seq Library (Vazyme) ^16^	S	Frozen	P	18	18	No
17	This study	Microarray	HumanHT-12 WG-DASL V4.0 (Illumina)	S	FFPE	P/M	11	20 ^17^	Yes (GSE196264)

MTC subtype based on somatic/germline *RET* mutation and/or family history. Abbreviations: S, sporadic; H, hereditary; U, unknown; P, primary tumor; M, metastasis; CNA, copy number alteration; WES, whole-exome sequencing; NA, not available. * Specifically refers to the approach reported in column 2. ** Based on authors’ data availability statement. ^1^ Two technical and four biological replicates were also tested. ^2^ Any data were found on www.ebi.ac.uk/arrayexpress, accessed on 6 April 2022 (based on several “Thyroid” related queries). ^3^ Tissue type (primary or metastasis) not specified. ^4^ Three technical replicates were also tested. ^5^ NGS panel with 50 genes. ^6^ Unknown; patients without data on *RET* germline mutations and/or MTC family history. ^7^ Different versions of the panel were used. ^8^ Somatic/germline *RET* mutational status predicted in silico. Unknown, patients with ambiguous or not available *RET* data. Metastases include 7 lymph nodes (LN) and 11 metastases from other sites. ^9^ Sporadic/hereditary type not specified. Metastases include eight LNs, one trachea, and one soft tissue metastasis. ^10^ Custom assay with at least 341 cancer-related genes. Mutation data deposition on http://cbioportal.org/msk-impact, accessed on 6 April 2022. ^11^ Authors’ indication of somatic mutations. Metastases include four LNs, two liver, and one chest wall metastasis.^12^ NGS panel with 17 thyroid-cancer-related genes. Novel mutation submission to COSMIC database. ^13^ Metastases include 33 LN metastases and 1 tumor recurrence. ^14^ NGS panel with 112 thyroid-cancer-related genes. ^15^ Metastases include three LN metastases and one LN recurrence. Three matched LN metastases were also tested. ^16^ RNAseq also includes 11 independent patients not profiled by WES. ^17^ Three non-neoplastic thyroids from three independent patients were also tested.

**Table 2:** Characteristics of the eight proprietary sporadic MTC patients.

Patient ID	Sex	Age (at dx)	TNM	Stage	ETE	Array ID (Primary Tumor)	Array ID (LNM)	*RET/RAS* Mutation	*RET* Polymorphisms
1	M	61	pT1aN1a	III	no	P 1	M 1	*RET* C630R	rs1799939; rs1800863 **
2	M	60	pT3N1b	IVa	yes	P 2	M 2	*RET* D898_E901del ^a^	wt
3	M	56	pT2mN1b	IVa	no	P 3	M 3.1; M 3.2 *	*RET* M918T ^b^	rs1800861
4	F	52	pT4N1b	IVa	yes	P 4	M 4	*HRAS* Q61K	rs1800861
5	M	50	pT3mN1b	IVa	yes	P 5	M 5 *	*RET* D898_E901del ^c^	rs1799939; rs1800863
6	M	28	pT3mN1b	IVa	yes	P 6	M 6	*RET* M918T	wt
7	F	49	pT3NX	III	yes	P 7	M 7 *	*RET* M918T	rs1799939; rs1800863 **
8	M	41	pT3N1b	IVa	no	P 8	M 8	*RET* E632_L633del	wt

Abbreviations: dx, diagnosis; ETE, extra thyroid extension; LNM, lymph node metastasis. * Three metachronous LNMs derived from subsequent lymph node dissection. Mean interval from initial surgery: 20.3 months (patient 3: 24 months, patient 5: 14 months, and patient 7: 23 months); in this interval, patients did not receive any systemic treatment and/or radiotherapy. ** Homozygosis for both polymorphisms. ^a^ *RET* del identified in subsequent testing following its original reporting [15]. ^b^ *RET* M918T mutation detected at low frequency in all the specimens tested for this patient. ^c^ *RET* del data available only for LNM; primary tumor data not evaluable (low-quality DNA).

## Data Availability

Raw and preprocessed gene expression data presented in this study are available in the NCBI Gene Expression Omnibus (GEO) repository with the accession number GSE196264.

## Supplementary Materials

The following supporting information can be downloaded at: https://www.mdpi.com/article/10.3390/cancers14081951/s1, Figure S1: Gene expression profiles and gene set enrichment analysis (GSEA) in our sporadic MTC cohort; Figure S2: Assessment of immune- and MTC-related gene sets in our sporadic MTC cohort; Figure S3: Assessment of selected MTC-related genes derived from the literature; Table S1: List of 1666 significant genes from MTC vs. NT class comparison (absolute FC > 2; FDR < 0.05); Table S2: List of gene sets significantly enriched in GSEA with C2-Canonical pathways collection (FDR < 0.05); Table S3: MEN2B/MEN2A signature gene list.
