# Supplementary material for: Medullary Thyroid Carcinoma Mutational Spectrum Update and Signaling-Type Inference by Transcriptional Profiles: Literature Meta-Analysis and Study of Tumor Samples
Source: Cancers (Basel). 2022 Apr 13;14(8):1951. doi: 10.3390/cancers14081951 (PMC9028774; doi:10.3390/cancers14081951)
Supplement: Supplementary file 1 [file cancers-14-01951-s001.zip › cancers-1645687-supplementary/Suppl.Figures.pdf]

Figure S1

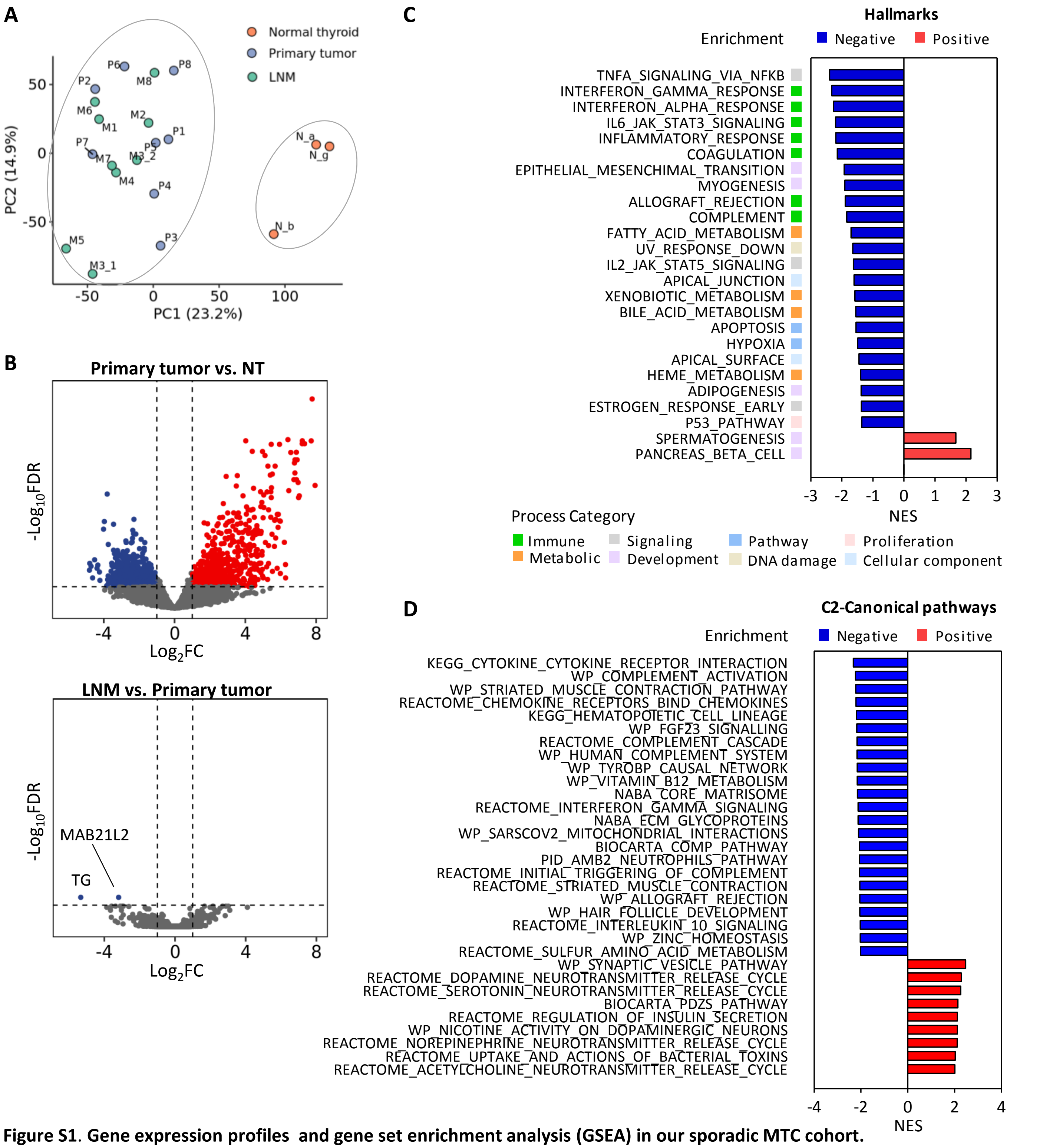

Figure S1. Gene expression profiles and gene set enrichment analysis (GSEA) in our sporadic MTC cohort. (**A**) Principal component analysis (PCA) in the proprietary series of 17 sporadic MTCs (patient matched primary tumors and lymph node metastases, LNMs) and 3 non-neoplastic thyroid (NT) controls. (**B**) Volcano plot of differentially expressed genes in the indicated comparisons. Log2 fold change (FC) and -log10 false discovery rate (FDR) values are shown; dashed lines represent selection cutoffs (absolute FC>2; FDR<0.05). Up- and down-regulated genes are shown in red and blue, respectively. The TG (Entrez 7038) and MAB21L2 (Entrez 10586) genes are indicated. (**C**) Significantly enriched gene sets (FDR<0.05) from GSEA Hallmarks collection. The process categories defined by Hallmarks collection curators are also shown. (**D**) Significantly enriched gene sets (FDR<0.05) from GSEA C2-Canonical pathways collection. GSEA performed with MTC vs. NT comparison data (Figure 4). Barplots show the normalized enrichment score (NES). Negatively and positively enriched gene sets are shown in blue and red, respectively. For (D) only gene sets with absolute NES>2 are shown; complete list available in Table S2.

Figure S2

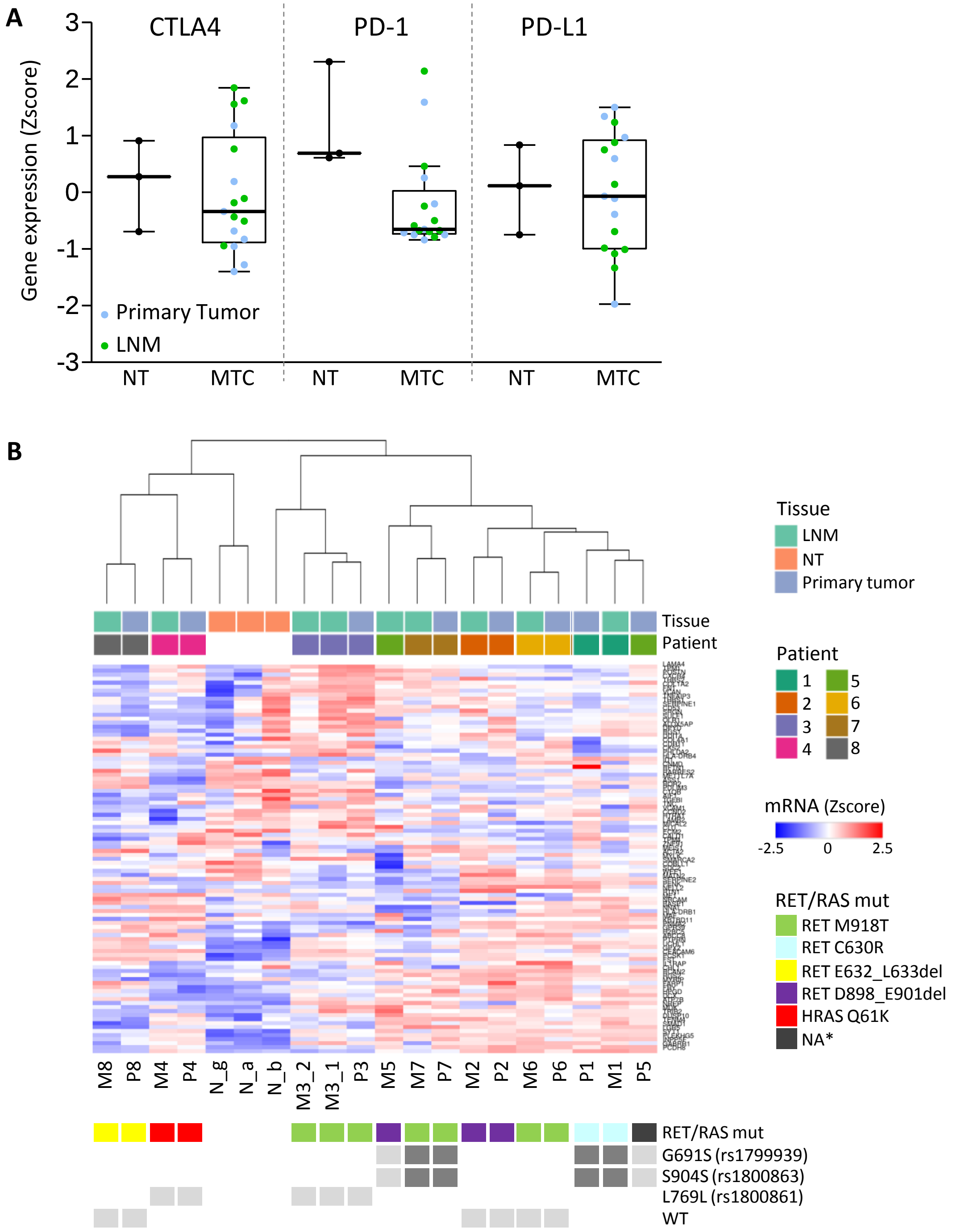

Figure S2. **Assessment of immune and MTC related gene sets in our sporadic MTC cohort.**
(**A**) Boxplot with scatterplot showing mRNA expression of the indicated immune related genes in the proprietary series of 17 sporadic MTCs and 3 non-neoplastic thyroid (NT) controls. MTC tissue types (primary tumors and lymph node metastases, LNMs) are highlighted by different colors; mRNA levels are shown as normalized data (Zscore). (**B**) Unsupervised hierarchical clustering analysis using the MEN2B/MEN2A signature; complete gene list also reported in Table S3. The features related to tissue type, patient ID and RET/RAS mutation (mut) with *RET* SNPs are indicated.

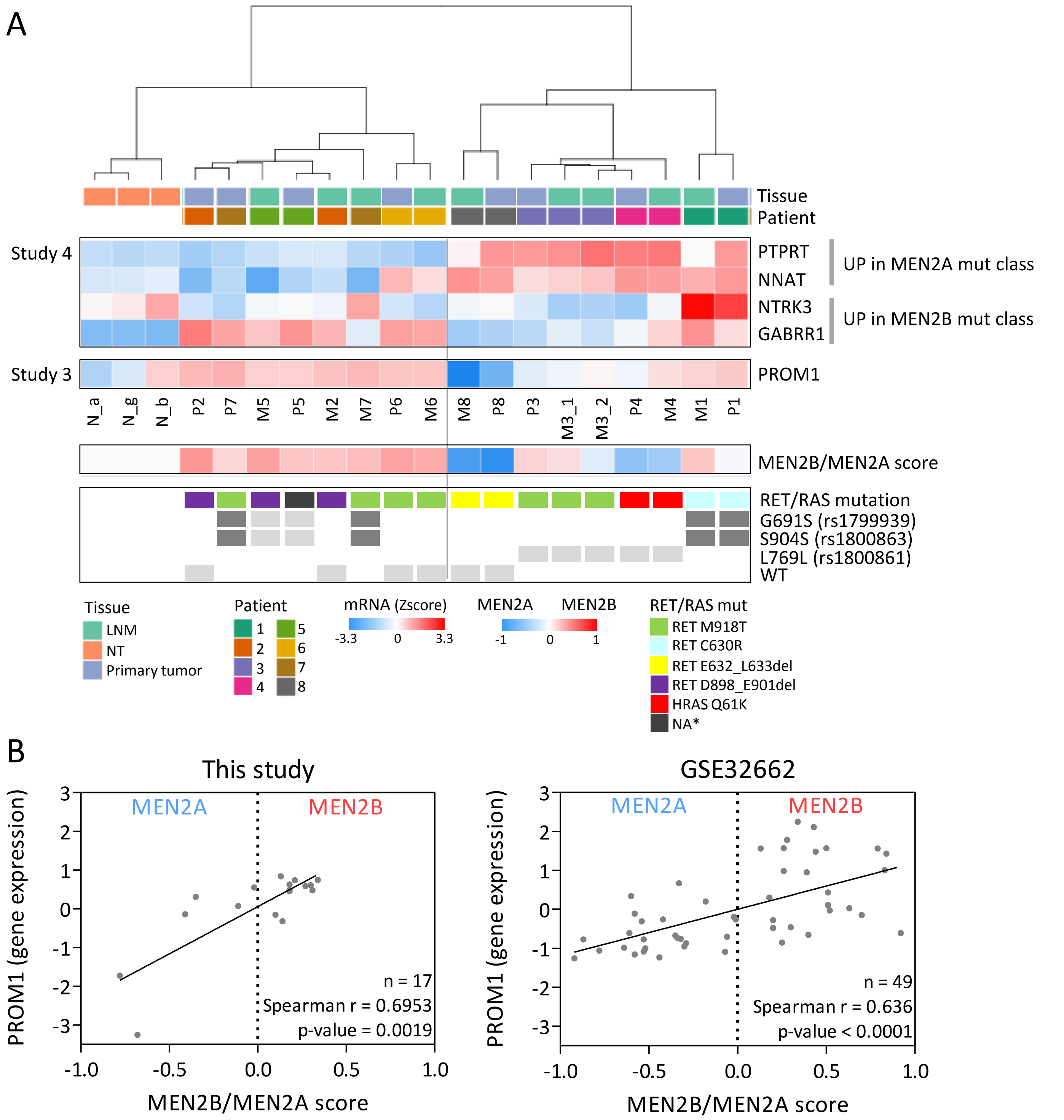

Figure S3. **Assessment of selected MTC related genes derived from literature.**
(**A**) Unsupervised hierarchical clustering analysis in our sporadic MTC cohort, using the 4-gene classifier reported by Oczko-Wojciechowska et al (Table 1, study 4). The expression of *PROM1* gene reported by Maliszewska et al. (Table 1, study 3) in the same samples is also shown. The sample corresponding tissue type, patient ID, MEN2B/MEN2A score, and *RET/RAS* mutation with *RET* SNPs are indicated. (**B**) Correlation by Spearman coefficient between *PROM1* gene and MEN2B/MEN2A score in our sporadic MTC cohort and in MTC from GSE32662 public series. The corresponding MEN2B/MEN2A signaling type derived from transcriptional score is indicated.
